# Mycobacterial Phenolic Glycolipids Selectively Disable TRIF-Dependent TLR4 Signaling in Macrophages

**DOI:** 10.3389/fimmu.2018.00002

**Published:** 2018-01-19

**Authors:** Reid Oldenburg, Veronique Mayau, Jacques Prandi, Ainhoa Arbues, Catherine Astarie-Dequeker, Christophe Guilhot, Catherine Werts, Nathalie Winter, Caroline Demangel

**Affiliations:** ^1^Unité d’Immunobiologie de l’Infection, INSERM U1221, Institut Pasteur, Paris, France; ^2^Université Paris Diderot, Paris, France; ^3^Institut de Pharmacologie et de Biologie Structurale (IPBS), Université de Toulouse, CNRS, UPS, Toulouse, France; ^4^Unité de Biologie et Génétique de la Paroi Bactérienne, Institut Pasteur, Paris, France; ^5^INRA, UMR 1282 Infectiologie et Santé Publique, Nouzilly, France; ^6^Université François Rabelais, Tours, France

**Keywords:** mycobacteria, phenolic glycolipids, macrophages, TRIF, TLR4, iNOS

## Abstract

Phenolic glycolipids (PGLs) are cell wall components of a subset of pathogenic mycobacteria, with immunomodulatory properties. Here, we show that in addition, PGLs exert antibactericidal activity by limiting the production of nitric oxide synthase (iNOS) in mycobacteria-infected macrophages. PGL-mediated downregulation of iNOS was complement receptor 3-dependent and comparably induced by bacterial and purified PGLs. Using *Mycobacterium leprae* PGL-1 as a model, we found that PGLs dampen the toll-like receptor (TLR)4 signaling pathway, with macrophage exposure to PGLs leading to significant reduction in TIR-domain-containing adapter-inducing interferon-β (TRIF) protein level. PGL-driven decrease in TRIF operated posttranscriptionally and independently of Src-family tyrosine kinases, lysosomal and proteasomal degradation. It resulted in the defective production of TRIF-dependent IFN-β and CXCL10 in TLR4-stimulated macrophages, in addition to iNOS. Our results unravel a mechanism by which PGLs hijack both the bactericidal and inflammatory responses of host macrophages. Moreover, they identify TRIF as a critical node in the crosstalk between CR3 and TLR4.

## Introduction

Phenolic glycolipids (PGLs) are polyketide synthase products that are only synthesized by a subset of pathogenic mycobacteria, including the W-Beijing family of *Mycobacterium tuberculosis* strains and *Mycobacterium leprae* (1–3). In structure, these phenolphtiocerol dimycocerosates (DIMs) share a common phenolic lipid backbone that is decorated with species-specific oligosaccharide moieties (Figure S1 in Supplementary Material). PGL from *M. tuberculosis* (PGL-tb) inhibited the inflammatory cytokine responses of mycobacteria-infected macrophages, suggesting that it mediates the virulence of W-Beijing strains by suppressing host innate immune responses (4). While the association between PGL-tb and mycobacterial virulence later appeared more complex, the anti-inflammatory activity of PGL-tb was confirmed, using naturally deficient *M. tuberculosis* strains that were genetically engineered to express PGL-tb (5). In line with these results, synthetic analogs of PGL-tb and *M. leprae* PGL-1 inhibited toll-like receptor (TLR)2-driven production of inflammatory cytokines and nitric oxide (NO) by macrophages (2, 6, 7). Since PGL-1 bound to immobilized TLR2 in solid-phase assays, it was proposed that PGL-1 and PGL-tb can act as TLR2 antagonists (2). Whether this mechanism is sufficient to explain the cytokine production defects of macrophages infected with PGL-expressing mycobacteria was not addressed.

In parallel, it was reported that recombinant *Mycobacterium bovis* BCG (rBCG) expressing PGL-1 instead of its native PGL (PGL-bov) exploit complement receptor (CR)3 for invasion of macrophages (2, 8). CR3, also known as Mac-1, CD11b/CD18, and αMβ2 integrin, is a widely expressed heterodimeric surface receptor, which in macrophages contributes to microbial pattern recognition and phagocytosis. CR3 is known to mediate the opsonic and non-opsonic uptake of *M. tuberculosis* and *M. leprae* by macrophages (9–11), *via* its complement-binding I-domain and its carbohydrate-binding lectin domain, respectively (12, 13). Based on biochemical evidence, the increased infectivity of PGL-1-expressing BCG was attributed to a selective interaction between its trisaccharide moiety and the lectin domain of CR3 (2). Of note, PGL-1-mediated phagocytosis required the Src-family kinase Lyn, a known mediator of β_2_-integrin signal transduction in macrophages (2, 14). In addition to promote macrophage invasion, PGL-1 increased the long-term survival of BCG within macrophages by a mechanism that remained unclear (8).

In the present work, we sought to determine if and how PGLs interfere with the bactericidal functions of macrophages. We found that PGLs limit the capacity of activated macrophages to induce nitric oxide synthase (iNOS) and generate NO upon mycobacterial infection, by downregulating the TLR4 adapter TIR-domain-containing adapter-inducing interferon-β (TRIF). In addition to suppressing iNOS production, PGLs decreased the TLR4-induced production of TRIF-dependent cytokines and chemokines. Our results thus provide a mechanism for both the immunomodulatory and virulence properties of PGLs. They support the general concept that PGL production was evolved by pathogenic mycobacteria to enhance intracellular survival and immune evasion.

## Materials and Methods

### Reagents

PGL-bov and DIMs were purified from bacterial cell pellets of *M. bovis* BCG and *Mycobacterium canettii*, respectively, as previously described (2). PGL-tb from *M. canettii* (#NR-36510) and PGL-1 from *M. leprae*-infected armadillos (#NR-19342) were obtained from BEI resources (https://www.beiresources.org/). Working solutions of lipids were prepared as follows: PGLs were dissolved in ethanol; DIMs were first recovered in a small volume of chloroform, before being added to water, then sonicated until complete suspension. These solutions were diluted >200 times in cell culture medium for cellular assays and compared to equivalent volumes of vehicle. Ultrapure LPS from *E. coli*, serotype O55:B5, TLR grade (#ALX-581-013-L001) was purchased from Enzo Life Sciences. Recombinant mouse IFN-γ (#PMC4031) and TNF-α (#PMC3014) were purchased from ThermoFisher Scientific. InSolution™ PP2 Src inhibitor (#529576) and ALLN (#208719) were from Calbiochem Merck Millipore, and chloroquine diphosphate salt (#C6628) from Sigma.

### Cell Cultures

Bone marrow-derived macrophage (BMDM) progenitors were obtained by flushing mouse femurs and tibias, followed by erythrocyte lysis with red blood cell lysis buffer (#B00003, Roche). BMDMs were obtained by a 7-day differentiation of progenitors in RPMI 1640 GlutaMAX™ medium (#61870-010, ThermoFisher Scientific) supplemented with 10% heat-inactivated fetal calf serum (#A15-102, PAA) and 10% L929-conditioned medium as a source of M-CSF [hereafter called complete medium (CM)]. THP-1 human monocytes (ATCC, TIB-202) were cultured in RPMI supplemented with 10% heat-inactivated fetal calf serum, penicillin, and streptomycin (#15140122, ThermoFisher Scientific). They were differentiated into macrophages by addition of 2 ng/ml phorbol 12-myristate 13-acetate (#P8139, Sigma) for 3 days.

### Mycobacteria Cultures and Cell Infection

Methods used to generate the PGL-expressing rBCGs used in this study and experimental validation that they grow comparably and express equivalent amounts of heterologous PGLs were reported previously (2, 8). Bacteria were grown at 37°C in suspension in Middlebrook 7H9 broth (#M0178) supplemented with ADC (#M0553) and 40 µg/ml kanamycin (#K0254), all from Sigma. Mycobacterial suspensions destined to infect BMDMs were pelleted at (3,200 × *g*) for 7 min, washed twice with phosphate-buffered saline (PBS), suspended in 5 ml of PBS before dissociation in M-tubes using the gentleMACS Dissociator (Miltenyi Biotec), then diluted in RPMI to the appropriate concentration. Before infection, BMDMs were washed twice with warm RPMI, followed by a 2 h infection in RPMI. BMDMs were washed twice again with warm RPMI, then incubated in complete CM.

### NO Quantification

Bone marrow-derived macrophages were cultured at 2 × 10^5^ cells per well in black clear-bottomed cell culture microplates (Greiner Bio-One International) for 24 h and infected or treated as indicated. Cells were washed once with warm PBS before addition of 5 µM 4,5-diaminofluorescein diacetate (#D225, Sigma), a cell permeable fluorescent dye for NO detection (15, 16), and incubation at 37°C in the dark. Fluorescence was measured using a fixed gain setting each hour for 5 h using a BMG FLUOstar OPTIMA Microplate reader (BMG Labtech) with emission and excitation wavelengths of 485 and 520 nm, respectively. In order to normalize cell number, BMDMs were subsequently stained with 0.05% crystal violet (#C0775, Sigma) in 2% ethanol for 15 min followed by four washes with PBS. Dye was then dissolved in methanol and absorbance was measured at 550 nm. All values were set as a fold change ratio to averaged value of unstimulated group.

### Flow Cytometry

Adherent mouse BMDMs were detached with accutase (#A6964, Sigma) for 20 min at 37°C and blocked with FcR Blocking reagent (#130-092-575, Miltenyi Biotec) for 15 min at 4°C. Antibodies used in flow cytometry were anti-CD11b (M1/70), anti-CD11c (HL3), anti-CD86 (GL1), anti-CD40 (HM40-3) from BD Biosciences; anti-TLR4 (MTS510) from Biolegend; anti-IFNGR1 (2E2) from eBioscience; anti-iNOS (M-19) from SCT and anti-goat AlexaFluor 647 Donkey (polyclonal IgG H&L) from Abcam (#ab150131). For intracellular staining of iNOS, macrophages were fixed with BD Lyse/Fix solution (#558049) for 10 min at 37°C, then permeabilized with BD Perm Buffer III (#558050) for 20 min at 4°C prior to antibody staining. Flow cytometric acquisitions were performed on a BD FACS Accuri C6 and data were analyzed using FlowJo software.

### Immunoblot Analysis and ELISA

Bone marrow-derived macrophages (1–3 × 10^6^) were washed and scraped in cold PBS, centrifuged, and then lysed in ice cold lysis buffer (20 mM Tris, 150 mM NaCl, 1mM EGTA, 1mM MgCl_2_, 1% *n*-Dodecyl-(β)-d-maltoside (#D4641), 4 mM sodium orthovanadate (#S6508), 50 mM NaF (#S6776), 10 µg/ml leupeptin (#L2884), 10 µg/ml aprotinin (#10820), 1 mM Pefabloc-sc (A8456)), all purchased from Sigma, for 15 min. Protein concentration was quantified with NanoDrop Light SpectroPhotometer (Thermo Fisher Scientific). Cell lysates were resolved on NuPAGE Bis-Tris gels and transferred to nitrocellulose membranes (ThermoFisher Scientific). Protein detections used the following antibodies: MyD88 (#3699), Phospho-Src (Tyr416, #2101), GAPDH (#2118), all from CST, and Ticam-1 (TRIF, #657102, Biolegend). Detection of pSrc (MW 60 kDa), MyD88 (MW 33 kDa), TRIF (MW 98 kDa), and GAPDH (MW 37 kDa) was performed in a single Western blot assay using multiple antibodies. Before using this technique, we verified that our antibodies were specific (data not shown). When only TRIF and GAPDH were analyzed, blots were sliced horizontally after transfer, then stained separately in order to capture images at optimal exposure times. Protein complexes were revealed with the ECL Prime detection reagent (GE Healthcare) and chemiluminescence reading on a Fuji LAS-4000 Luminescent Image Analyzer. IFN-β and TNF-α concentrations in BMDM culture supernatants were measured by ELISA (Biolegend, #439407 and #430901), following the manufacturer’s protocol.

### RNA Extraction and Real-time Quantitative RT-PCR

Qiagen RNeasy Mini Kit (#74104) was used to extract total RNA from BMDMs. cDNA was amplified from 1 µg of total RNA using the high-capacity cDNA reverse transcription kit with added RNAse inhibitor (#4374966, Applied Biosystems). Relative mRNA levels were quantified by qRT-PCR using power SYBR green and with gene-specific primers (Table 1). Amplification conditions and dissociation step were as follows: 50°C for 2 min, 95°C for 10 min followed by 40 cycles (95°C for 15 s and 60°C for 1 min). ABI 7300 Sequence Detection System (Applied Biosystems) was used for data acquisition. Fold increase values were calculated for each gene transcript using the 2^−ΔΔCt^ method, using RPL19 as a house-keeping gene.

**Table 1:** Primers used in the qRT-PCR analysis.

Gene	Primers (5′–3′)
*Nos2* (17)	ACATCGACCCGTCCACAGTAT
	CAGAGGGGTAGGCTTGTCTC
*Arg1* (17)	CTCCAAGCCAAAGTCCTTAGAG
	AGGAGCTGTCATTAGGGACATC
*Cxcl10* (18)	GGATCCCTCTCGCAAGGA
	ATCGTGGCAATGATCTCAACA
*Ifnb1* (19)	CCCTATGGAGATGACGGAGA
	ACCCAGTGCTGGAGAAATTG
*Il6* (20)	AGTTGCCTTCTTGGGACTGA
	TCCACGATTTCCCAGAGAAC
*Cebpb* (21)	GGAGACGCAGCACAAGGT
	AGCTGCTTGAACAAGTTCCG
*Tnf* (17)	CTGGGACAGTGACCTGGACT
	GCACCTCAGGGAAGAGTCTG

### Mice

C57BL/6J (JAX™) and Itgam^−/−^ (B6.129S4-Itgamtm1Myd/J) mice were obtained from Charles River and Jackson Laboratories, respectively. TRIF^LPS2/LPS2^ mice [C57BL/6J—Ticam1^Lps2^ (22)] were originally from B. Beutler et al. (The Scripps Research Institute, CA, USA) and back-crossed into the C57BL/6J background at Institut Pasteur. TLR2^−/−^ [B6.Cg-Tlr2^tm1Aki^ (23)] and MyD88^−/−^ [B6.129-Myd88^tm1Aki^ (24)] mice were obtained from S. Akira (Osaka University, Japan). All animals were bred and housed under pathogen-free conditions in our animal facilities with food and water *ad libitum*. Transgenic mice were used between 6 and 10 weeks of age, with age and sex-matched wild-type controls. Since we only used mice as a source of bone marrow, the described experiments did not require approval from the French Ministry of Higher Education and Research. They were performed in compliance with the European Communities Council Directive of 22 September 2010 on the approximation of laws, regulations, and administrative provisions of the Member States regarding the protection of animals used for scientific purposes.

### Statistical Analysis

Statistical analyses were performed with the *StatView*^®^ 5 software (SAS Institute, Inc.). The Prism software (5.0d) was used for graphical representation.

## Results

### PGLs Inhibit Infection-Induced Production of iNOS and NO in Activated Macrophages

**Figure 1:**
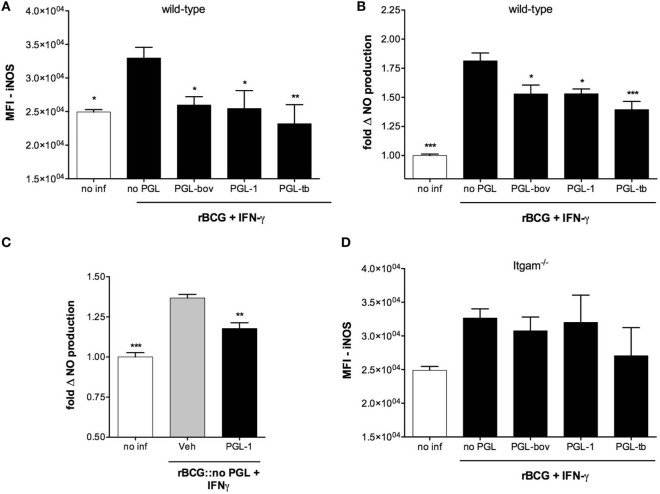
Phenolic glycolipids (PGLs) inhibit infection-induced production of iNOS and NO in activated macrophages. **(A)** Differential induction of iNOS in bone marrow-derived macrophage (BMDMs) infected with rBCG:no PGL, rBCG:PGL-bov, rBCG:PGL-1 or rBCG:PGL-tb, or non-infected BMDMs (no inf), as determined by intracellular detection of iNOS using flow cytometry. BMDMs were infected for 2 h at a MOI of 1:1, then washed and cultivated in the presence of 100 U/ml IFN-γ for 48 h. Data are mean fluorescence intensities (MFI) ± SEM (*n* = 3). **(B)** Differential production of NO in the same conditions as in **(A)**. Data are mean NO levels ± SEM (*n* = 8), expressed as fold changes relative to non-infected controls (no inf). **(C)** NO production by BMDMs pretreated with 25 µM PGL-1 or ethanol vehicle (Veh) for 24 h prior to 48 h of infection with rBCG:no PGL at a MOI of 1:1 in the presence of 100 U/ml IFN-γ. **(D)** as in **(A)** in Itgam^−/−^ BMDMs. Data shown are from one experiment repeated twice with similar results. *P < 0.05, **P < 0.01, ***P < 0.001, following statistical comparison by repeated measures ANOVA with Tukey *post hoc* test, relative to rBCG:no PGL.

Following infection with mycobacteria and the early activation of innate immunity, T cells are stimulated to produce IFN-γ. This cytokine induces the expression of iNOS in infected macrophages, with subsequent production of NO efficiently controlling the growth of intracellular bacteria (25–28). To see if PGLs affect NO production in activated macrophages, we compared intracellular levels of iNOS in BMDMs infected with recombinant BCG expressing PGLs (rBCG:PGLs), or PGL-deficient BCGs (rBCG:no PGL) as control, in the presence of IFN-γ. Figure 1A shows that rBCG:bov, rBCG:PGL-1, and rBCG:PGL-tb all elicited less iNOS than rBCG:no PGL in infected macrophages. Consistently, the production of NO was lower in cells infected with any of the PGL-expressing strains, compared to those infected with PGL-deficient rBCG (Figure 1B). Importantly, addition of soluble PGL-1 to BMDMs was sufficient to reduce the cell production of NO upon infection with PGL-deficient rBCG (Figure 1C).

Since PGL-1 was previously reported to interact with CR3, we tested the potential involvement of this receptor in PGL-mediated inhibition of iNOS and NO production, using CD11b-deficient (Itgam^−/−^) macrophages. PGL-expressing rBCGs induced comparable production of iNOS as PGL-deficient BCG in Itgam^−/−^ macrophages, implying that CR3 is involved (Figure 1D). Together, these data suggested that PGLs have the intrinsic capacity to suppress the infection-induced production of NO in activated macrophages, by a mechanism involving CR3.

### PGLs Reduce the LPS/IFN-γ-Induced Production of iNOS in a CR3-Dependent Manner

**Figure 2:**
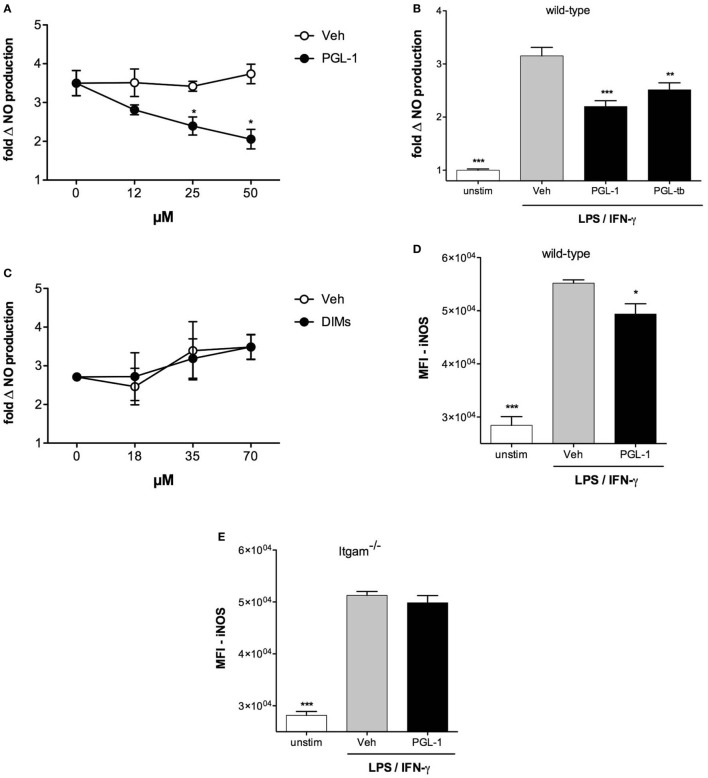
Phenolic glycolipids (PGLs) reduce LPS/IFN-γ-induced production of iNOS in a CR3-dependent manner. **(A)** Differential production of NO by bone marrow-derived macrophages (BMDMs) pretreated with increasing concentrations of PGL-1 or vehicle (Veh) for 24 h prior to a 24 h stimulation with 1 µg/ml LPS and 100 U/ml IFN-γ. Data are mean NO levels ± SEM (*n* = 4), expressed as fold changes relative to non-treated, stimulated controls. *P < 0.05, Mann–Whitney test comparing PGL-1-treated to vehicle controls at each concentration. **(B)** As in **(A)** with 25 µM PGL-1 or 25 µM PGL-tb; unstim: non-treated, non-stimulated controls. **(C)** As in **(A)**, with increasing concentrations of dimycocerosates (DIMs). **(D)** Differential induction of iNOS in BMDMs exposed to 25 µM PGL-1 or vehicle (Veh) for 24 h prior to a 24 h stimulation with LPS/IFN-γ. Controls are non-treated, non-stimulated cells (unstim). Data are mean fluorescence intensities (MFI) ± SEM (*n* = 3). **(E)** As in **(D)** in Itgam^−/−^ BMDMs. Data shown are from one experiment repeated twice with similar results. Statistical comparisons in **(B,D,E)** were performed with repeated measures ANOVA with Tukey *post hoc* test, relative to the vehicle-treated, LPS/IFN-γ-stimulated group. *P < 0.05, **P < 0.01, ***P < 0.001.

Induction of iNOS requires the cooperative activation of the JAK–STAT and pattern recognition receptor signaling pathways (29). To gain insight into the mechanism by which PGLs downregulate iNOS in mycobacteria-infected macrophages, we next tested if PGLs affected NO production in BMDMs stimulated with IFN-γ and the TLR4 agonist LPS. PGL-1 added to macrophages at a concentration superior to 12 µM prior to stimulation with LPS/IFN-γ indeed caused a dose-dependent reduction in NO production (Figure 2A). Notably, NO decrease was not observed if cells were treated with PGL-1 at the time of LPS/IFN-γ stimulation, and required a preincubation with PGL-1 of at least 6 h (Figure S2A in Supplementary Material). PGL-tb was equivalent to PGL-1 in its capacity to decrease the LPS/IFN-γ-stimulated production of iNOS by BMDMs (Figure 2B). In contrast, phthiocerol DIMs, which correspond to PGLs devoid of phenol ring and oligosaccharide domains (Figure S1 in Supplementary Material) had no inhibitory effect on NO production in the same conditions of cell pretreatment and stimulation (Figure 2C). This indicated that the lipid backbone of PGLs is not sufficient to inhibit the LPS/IFN-γ-induced production of iNOS.

Consistent with our data in Figure 1, the inhibitory activity of PGL-1 on LPS/IFN-γ-mediated production of NO correlated with a significant decrease in iNOS (Figure 2D) and required BMDM expression of CR3 (Figure 2E). Since PGL-1 binds to TLR2 *in vitro* and decreases the TLR2-induced production of cytokines and NO by human macrophages (2, 6, 7, 30), we next tested if TLR2 was involved in the observed effects. Decreased production of iNOS and NO was maintained in PGL-1-treated, LPS/IFN-γ-stimulated TLR2-deficient BMDMs (Figures S2B,C in Supplementary Material), ruling out this possibility. We concluded that PGLs suppress the LPS/IFN-γ-induced induction of iNOS in a CR3-dependent and TLR2-independent manner.

### PGLs Impair TLR4-Mediated Downstream Signaling Pathways

**Figure 3:**
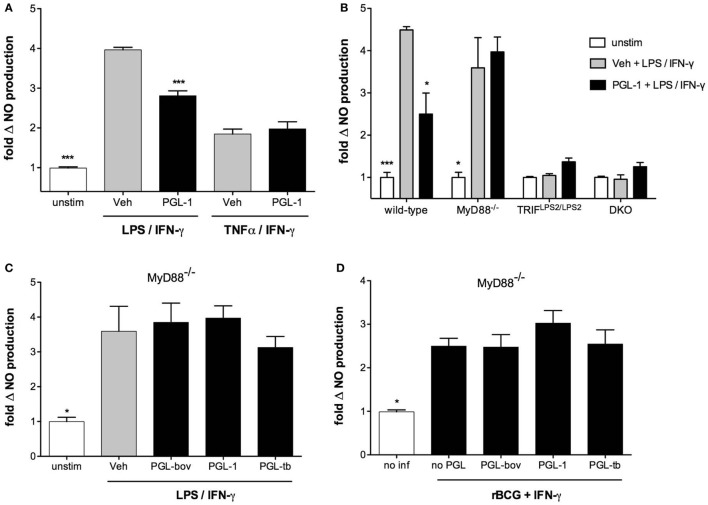
Phenolic glycolipids (PGLs) impair TLR4-mediated downstream signaling pathways. **(A)** Differential production of NO by bone marrow-derived macrophages (BMDMs) pretreated with 25 µM PGL-1 or vehicle (Veh) for 24 h prior to a 24 h stimulation with 1 µg/ml LPS + 100 U/ml IFN-γ, or 10 ng/ml TNF-α + 100 U/ml IFN-γ. ***P < 0.001, Mann–Whitney test, comparing PGL-1-treated to vehicle controls in each condition of cell stimulation. **(B)** Differential production of NO by wild-type, MyD88^−/−^, TRIF^Lps2/Lps2^, or DKO BMDMs treated with 25 µM PGL-1 or vehicle (Veh) for 24 h prior to a 24 h stimulation with 1 µg/ml LPS + 100 U/ml IFN-γ. Controls include non-treated, non-stimulated cells (unstim). **(C)** Differential production of NO by MyD88^−/−^ BMDMs treated with 25 µM PGL-bov, 25 µM PGL-1, 25 µM PGL-tb, or vehicle (Veh) for 24 h prior to a 24 h stimulation with LPS/IFN-γ. **(D)** Differential production of NO by MyD88^−/−^ BMDMs infected with rBCG:no PGL, rBCG:PGL-bov, rBCG:PGL-tb, or rBCG:PGL-1 at a MOI of 5:1, or non-infected (no inf). In **(B–D)**, data are mean NO levels ± SEM (*n* ≥ 3), expressed as fold changes relative to non-stimulated (unstim) or non-infected (no inf) controls. Data shown are from one experiment repeated twice with similar results. *P < 0.05, **P < 0.01, ***P < 0.001, repeated measures ANOVA with Tukey *post hoc* test, relative to vehicle or no PGL controls.

We next sought to determine which of the TLR4 and IFN-γ receptor (IFNGR) signaling pathways was targeted by PGLs. BMDMs were stimulated with LPS/IFN-γ or TNF-α/IFN-γ, two combinations of reagents leading to significant production of NO. Figure 3A shows that PGL-1 only decreased the NO production of LPS/IFN-γ-stimulated cells, suggesting that PGLs interfere with TLR4 signaling independently of IFN-γ. Consistently, PGL-1 treatment did not alter the levels of total and Tyr701-phosporylated Stat1 in BMDMs stimulated with IFN-γ (data not shown). Surface expression of TLR4, IFN-γ receptor 1, or CD11b was not altered by PGL-1 treatment of BMDMs (Figure S3 in Supplementary Material), implying that PGL-1 interferes with signaling events downstream of TLR4. Since TLR4 uses two adaptor proteins (MyD88 and TRIF), we examined if they equally contribute to TLR4-induced production of NO. We used BMDMs generated from MyD88^−/−^, TRIF^Lps2/Lps2^, or double knock-out (DKO) mice. Figure 3B shows that LPS/IFN-γ-stimulated production of NO was strictly mediated by TRIF. Notably, PGL-1-mediated inhibition of LPS/IFN-γ-stimulated production of NO was lost in MyD88^−/−^ BMDMs (Figure 3B). Comparable findings were obtained with PGL-tb and PGL-bov (Figure 3C). In contrast to wild-type BMDMs (Figure 1A), infection of MyD88^−/−^ BMDMs with PGL-expressing rBCGs did not alter their relative production of NO (Figure 3D). Together, these data in Figure 3 thus suggest that PGLs decrease TRIF-dependent production of NO in a MyD88-dependent manner.

### PGL-1 Downregulates TRIF Protein Levels

**Figure 4:**
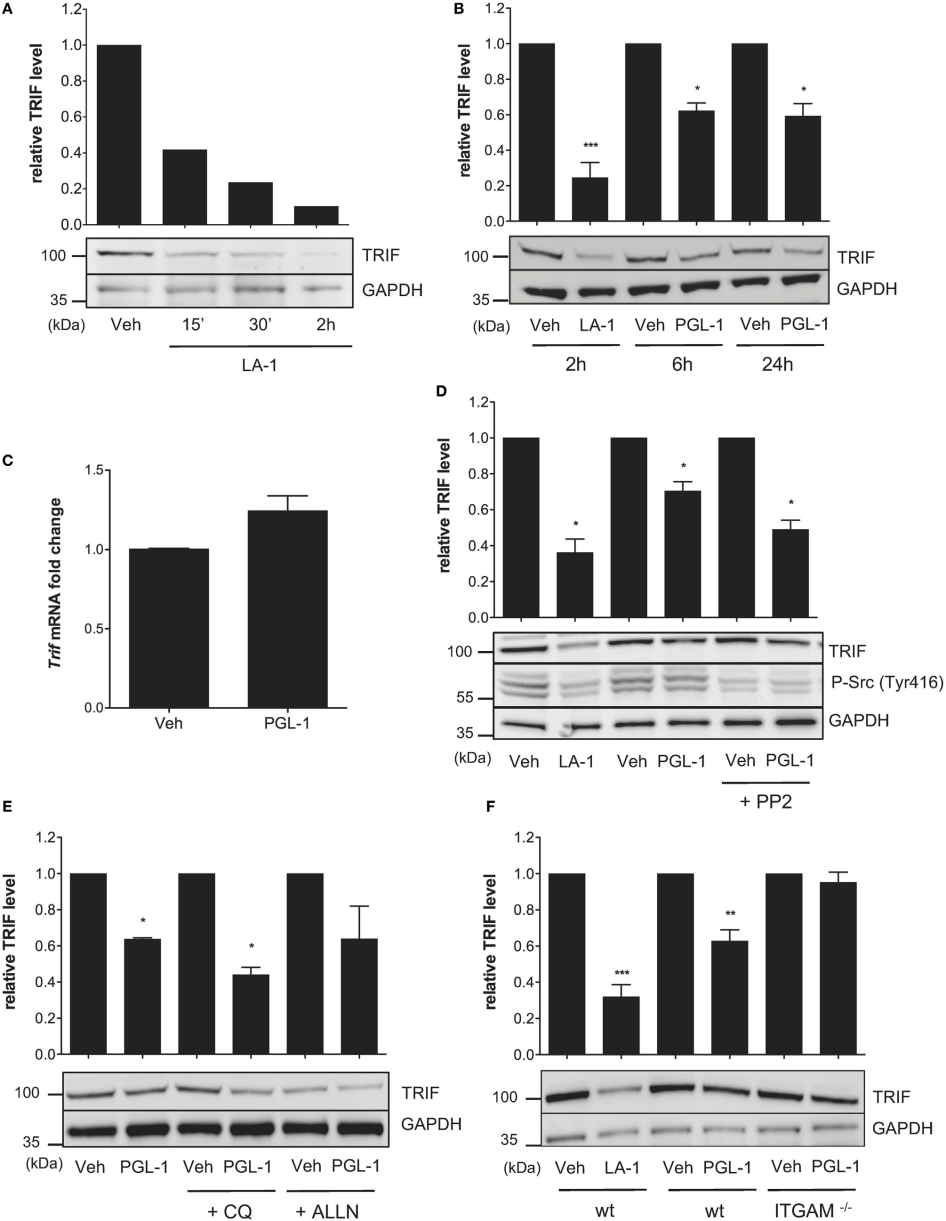
Phenolic glycolipid (PGL)-1 binding to CR3 downregulates TRIF protein levels. **(A)** Western blot analysis of TRIF with GAPDH as loading control, in bone marrow-derived macrophages (BMDMs) treated with 15 µM leukadherin (LA)-1 or DMSO vehicle (Veh) for the indicated times with relative quantification of TRIF levels. **(B)** Same as in **(A)**, for BMDMs treated for the indicated times with 15 µM LA-1 or 25 µM PGL-1, or their respective solvents (Veh). **(C)** Quantitation of *TRIF* mRNAs in BMDMs treated with 25 µM PGL-1 or vehicle (Veh) for 6 h. Data are mean transcript levels ± SEM (*n* = 4), expressed as fold changes relative to vehicle controls. They are representative from three independent experiments with similar results. **(D)** Relative TRIF levels in BMDMs treated with 15 µM LA-1 for 2 h, or 25 µM PGL-1 for 6 h in the presence or absence of 10 µM PP2 (Veh); **(E)** in BMDMs pretreated with 50 µM chloroquine (CQ) or calpain inhibitor (ALLN) for 2 h prior to a 24 h exposure to 25 µM PGL-1; **(F)** in wild-type (wt) or ITGAM^−/−^ BMDMs treated with 15 µM LA-1 for 2 h, or 25 µM PGL-1 for 24 h. Data in **(B,D,E,F)** are mean band intensities ± SEM from two to five independent experiments, with one representative blot picture.

TLR4-driven inside–out activation of CR3 was previously shown to dampen TLR4 signaling in BMDMs through a negative feedback mechanism involving Src-mediated degradation of MyD88 and TRIF by the ubiquitin:proteasome system (31). In parallel, it was reported that Leukadherin (LA)-1, a CR3 agonist, downregulates MyD88 protein levels in TLR7/8-stimulated THP-1 macrophages (32). We hypothesized that CR3 engagement by PGLs may affect TLR4 signaling in macrophages by interfering with endogenous levels of MyD88 and/or TRIF. Consistent with previous work, MyD88 levels were decreased in BMDMs treated with LA-1 (Figure S4A in Supplementary Material). Notably, the inhibitory activity of LA-1 on MyD88 levels was modest in comparison to that on TRIF. A 2 h treatment of BMDMs with 15 µM LA-1 indeed provoked >90% reduction in TRIF protein levels (Figure 4A). A comparable decrease was seen in human THP-1 macrophages treated with LA-1 (Figure S4B in Supplementary Material). Exposing BMDMs or THP-1 cells to 25 µM PGL-1 for >6 h led to similar effects, although maximal downregulation of TRIF by PGL-1 reached a plateau at 50% (Figure 4B; Figure S4B in Supplementary Material). In contrast, PGL-1 had no major effects on MyD88 levels (Figure S4C in Supplementary Material).

PGL-1 treatment did not alter the level of TRIF transcripts in BMDMs, indicating that PGL-1-mediated decrease in TRIF protein levels operates at a posttranscriptional level (Figure 4C). It was maintained in the presence of the Src family tyrosine kinase inhibitor PP2 (Figure 4D), suggesting that PGL-1 operates independently of Src activation. We tested if PGL-1-driven reduction in TRIF depended on lysosomal or proteasomal degradation using chemical inhibitors of these pathways, but no significant restoration of TRIF levels could be observed (Figure 4E). Notably, PGL-1-mediated decrease in TRIF was not detected in Itgam^−/−^ BMDMs, further illustrating the involvement of CR3 in this process (Figure 4F).

### PGL-1-Mediated Decrease in TRIF Impairs Downstream Signaling Events

**Figure 5:**
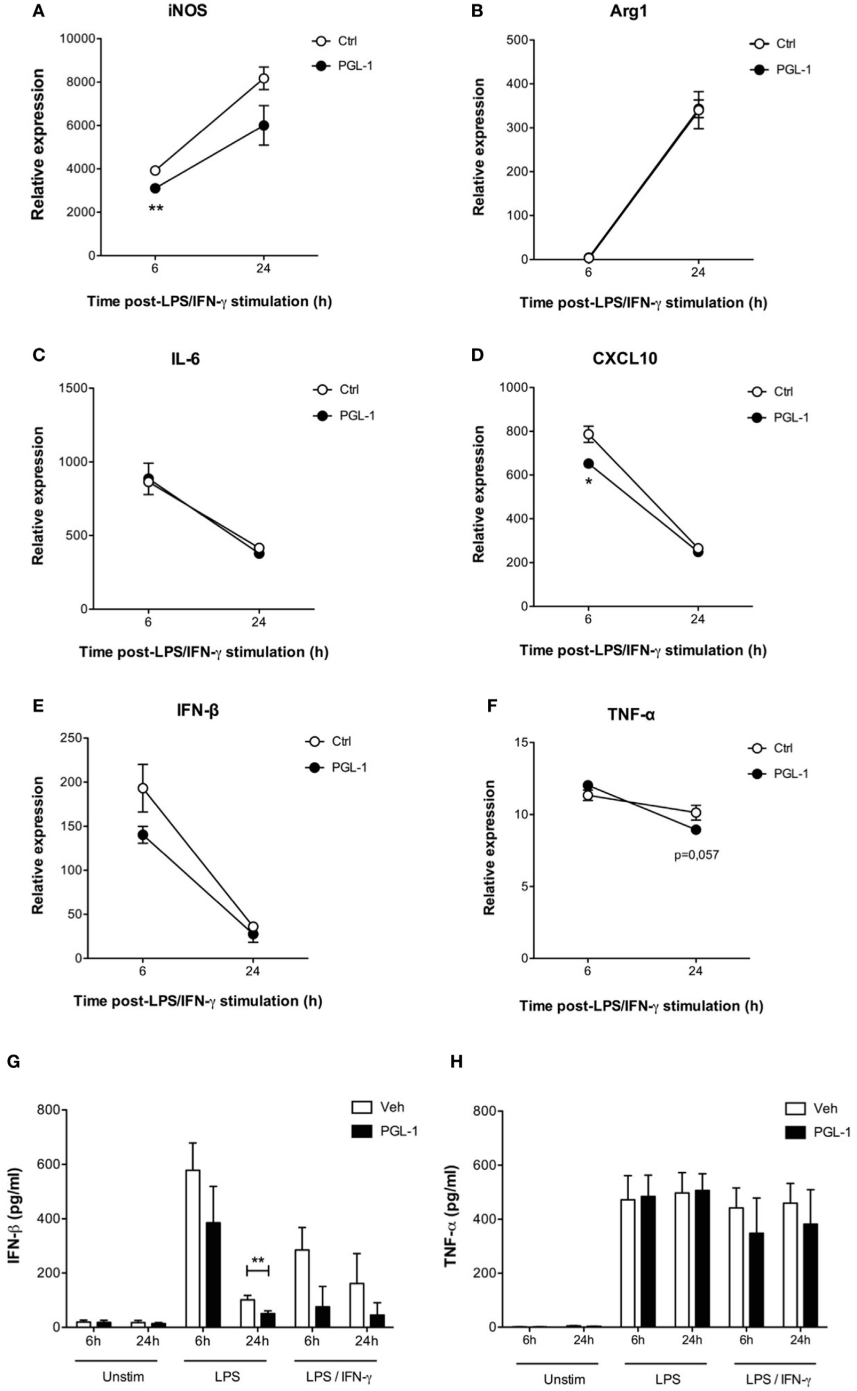
Phenolic glycolipid (PGL)-1-mediated decrease in TRIF impairs downstream signaling events. Quantitation of mRNAs in bone marrow-derived macrophages (BMDMs) treated with 25 µM PGL-1 or vehicle (Veh) for 24 h prior to 6–24 h of stimulation with LPS/IFN-γ: **(A)** *Nos2* (iNOS); **(B)** *Arg1* (Arginase 1); **(C)** *IL6* (IL-6); **(D)** *CXCL10* (CXCL10); **(E)** *Ifnb1* (IFN-β); **(F)** *Tnf* (TNF-α). Data are relative mean transcript levels (*n* > 3) ± SEM. *P < 0.05, **P < 0.01, Student’s *t*-test, relative to RPL19 house-keeping gene. They are representative of at least two independent experiments with similar results. Production of IFN-β **(G)** and TNF-α **(H)** by BMDMs pretreated with 25 µM PGL-1 for 24 h prior to 6–24 h of stimulation with LPS (*n* = 6) or LPS/IFN-γ (*n* = 2). Data in **(G,H)** are mean cytokine concentrations from biological replicates ± SEM. **P < 0.01, Mann–Whitney test comparing PGL-1-treated cells to vehicle controls.

We next investigated to what extent PGL-1-driven decrease in TRIF protein level altered TLR4 signaling events in activated macrophages. Consistent with our data in Figures 1 and 2, pretreating BMDMs with PGL-1 for 24 h prior to LPS/IFN-γ stimulation durably suppressed *Nos2* transcription (Figure 5A). Expression of Arginase 1 (*Arg1*), which competes with iNOS for arginine, does not depend on TRIF. Figure 5B shows that contrary to *Nos2*, the LPS/IFN-γ-induced transcription of *Arg1* was not modified by PGL-1 pretreatment. Similarly, expression of MyD88-dependent IL-6 (*Il6*) and M2-inducer CEBPB (*Cebpb*) in LPS/IFN-γ-stimulated BMDMs were not impacted by pre-exposure to PGL-1 (as shown for IL-6 in Figure 5C). In contrast, the level of CXL10 (*Cxcl10*) transcripts, which relies on TRIF-IRF3 signaling, was significantly reduced by PGL-1 pretreatment in BMDMs stimulated with LPS/IFN-γ for 6 h (Figure 5D). Although statistical significance was not reached, a similar trend was observed with the expression of the TRIF-dependent cytokine IFN-β (*Ifnb1*) (Figure 5E). By measuring IFN-β concentration in culture supernatants, we could confirm that PGL-1 pretreatment reduces significantly the ability of BMDMs to produce IFN-β in response to TLR4 stimulation (Figure 5G). In comparison, gene and protein expression of TNF-α (*Tnf*), which depend on both the MyD88 and TRIF pathways, were minimally affected (Figures 5F–H).

## Discussion

We report in the present work that exposure of macrophages to mycobacterial PGLs affects the integrity of their TLR4 signaling pathway. Using PGL-1 as a model, we show that PGLs operate by selectively downregulating the TLR4 adaptor TRIF. Since TRIF mediates the production of iNOS and selected cytokines and chemokines in macrophages, PGL production endows mycobacteria with the capacity to alter both the bactericidal and inflammatory responses of the host during chronic infection.

This property adds to the previously reported inhibitory activity of PGL-1 and PGL-tb on TLR2 signaling, which was evidenced by a lower induction of NF-κB and associated production of TNF-α in macrophages stimulated with Pam3CSK4 in the presence of PGLs (2, 6, 7). Notably, PGL-mediated inhibition of TLR2 signaling operated immediately and independently of CR3-mediated phagocytosis. This is in stark contrast with the observed effects of PGLs on TRIF-dependent TLR4 signaling, which required that macrophages express CR3 and are exposed to PGLs for >6 h. Using TLR2-deficient BMDMs, we excluded the possibility that TLR2 contributes to PGL-mediated inhibition of TLR4 signaling. Unlike TRIF, MyD88 was not impacted by macrophage pretreatment with PGL-1. Together, these data thus suggest that PGLs affect TRIF independently of their effect on TLR2 signaling. Pretreating macrophages with PGL-1 prior to stimulation with TLR3 ligand poly(I:C) did not alter their IFN-β production (Figure S5 in Supplementary Material), thus limiting the functional relevance of PGL-1-mediated decrease in TRIF to TLR4 signaling.

Mycobacteria display multiple TLR2 and TLR4 agonists, and studies using TLR knock-out animals have shown the importance of TLR2/4 signals in host responses to chronic mycobacterial infection, *via* production of bactericidal molecules and pro-inflammatory mediators (3, 33, 34). By inhibiting TLR2 signaling (previous work) and TRIF-dependent TLR4 signaling (present study) in macrophages, PGL production is thus likely to alter the immune control of *M. tuberculosis* and *M. leprae* infection *in vivo*. Interestingly, several strains of *M. tuberculosis* belonging to the W-Beijing lineage were shown to be potent activators of TLR4 and inducers of Type I IFNs in BMDMs (35). Production of Type I IFNs during infection with *M. tuberculosis* [reviewed in Ref. (36)] and *M. leprae* (37) is believed to promote rather than limit disease progression, through diverse autocrine and paracrine mechanisms contributing to suppress IFN-γ production and IFN-γ-induced microbicidal responses. However, recent studies have indicated that in conditions where IFN-γ signaling is absent, Type I IFNs confer protection against *M. tuberculosis* infection (38, 39). Our observation that PGLs limit the ability of macrophages to produce IFN-β upon TLR4 activation, irrespective of IFN-γ stimulation, is, therefore, particularly interesting to consider in the context of early immune responses to infection.

We excluded the possibility that PGLs suppress TRIF gene transcription or promote TRIF proteasomal or lysosomal degradation, but failed to identify a mechanism linking CR3 with TRIF protein loss. PGL-mediated inhibition of NO production was lost in MyD88^−/−^ macrophages, suggesting that TRIF degradation requires MyD88. Previous studies have shown that PGL-1 differs from other PGLs in capacity to promote the CR3-dependent uptake of mycobacteria by macrophages (2). Consistently, PGL-1 binding to CR3 was not displaced by oligosaccharides from other PGLs in solid-phase assays (2). Whether all PGLs can bind to a distinct region of the CR3 receptor, without activating downstream Src signaling, is unknown. Our observation that PGL-1, PGL-tb, and PGL-bov comparably suppress TLR4-induced induction of iNOS, in a CR3-dependent manner, supports this possibility. Alternatively, PGLs may bind to a distinct macrophage receptor, secondarily interfering with CR3. Incubation of BMDMs with PGL-1 did not alter their surface expression of TLR4 (Figure S3 in Supplementary Material) nor basal production of IFN-β (Figure 5G), arguing against a direct interaction between PGLs and TLR4. Further work will be needed to determine how CR3 and MyD88 connect with PGL-driven TRIF downregulation, and whether TRIF production is altered at the translational level, or if other mechanisms are at play. Recently, it was reported that PGL-1 expressed by recombinant *Mycobacterium marinum* induces BMDMs to produce enhanced levels of iNOS transcripts after 6 h of infection (40). It would be interesting to see if the production of NO is augmented in macrophages infected *M. marinum*:PGL-1, and if the stimulatory effect of PGL-1 persists beyond 6 h postinfection in this system. If so, this would suggest that the mycobacterial strain expressing PGL-1 influences its effect on iNOS production by infected macrophages.

Aside from macrophages, CR3 is expressed by monocytes, dendritic cells, neutrophils, NK cells, basophils, eosinophils, and platelets. Interestingly, CD11b was reported to regulate TLR4-induced signaling pathways in a positive manner in dendritic cells (41). The authors proposed that, in these cells, but not in macrophages, CD11b facilitates TLR4 endocytosis and subsequent TRIF-mediated signaling in endosomes. Our preliminary investigations in the mouse MutuDC line showed that pretreatment with PGL-1 inhibits the LPS-stimulated upregulation of CD86 at the cell surface (data not shown), which suggests that PGL-1 comparably affects TRIF-dependent TLR4 signaling in dendritic cells. In all, our findings expand the list of subtle functional alterations caused by mycobacterial PGLs in the biology of host macrophages, namely hijacking the CR3 receptor for increased infectivity and directly interacting with surface-displayed TLR2. In addition to improve our understanding of how PGLs manipulate innate immunity receptor signaling and communication, our findings reveal a novel element of crosstalk between TLR and the complement system that is exploited by pathogens to improve persistence in infected hosts (42).

## Author Contributions

RO, VM, CW, NW, and CD conceived the experiments. RO and VM conducted the experiments. JP, AA, CA-D, CG, and CW provided essential materials. All authors analyzed the results and revised the manuscript.

## Conflict of Interest Statement

The authors declare that the research was conducted in the absence of any commercial or financial relationships that could be construed as a potential conflict of interest.
