# Supplementary material for: Mycobacterial Phenolic Glycolipids Selectively Disable TRIF-Dependent TLR4 Signaling in Macrophages
Source: Front Immunol. 2018 Jan 19;9:2. doi: 10.3389/fimmu.2018.00002 (PMC5780341; doi:10.3389/fimmu.2018.00002)
Supplement: Supplementary file 1 [file Image_1.PDF]

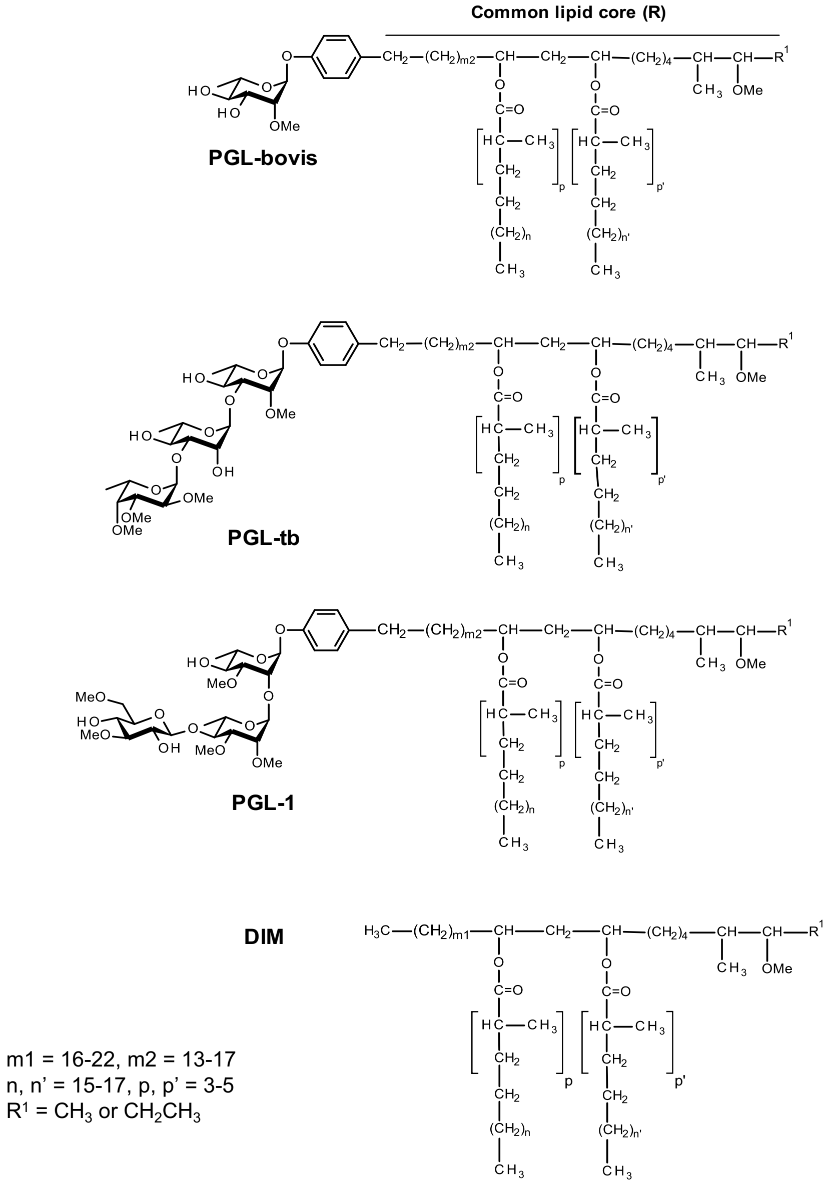

FIGURE S1: Structure of the major forms of PGLs made by *Mycobacterium bovis* (PGL-bovis), *M. tuberculosis* (PGL-tb) and *M. leprae* (PGL-1), and of mycobacterial DIMs. The common lipid core is composed of a long-chain β-diol esterified by polymethyl-branched fatty acids.
