# Supplementary material for: Mycobacterial Phenolic Glycolipids Selectively Disable TRIF-Dependent TLR4 Signaling in Macrophages
Source: Front Immunol. 2018 Jan 19;9:2. doi: 10.3389/fimmu.2018.00002 (PMC5780341; doi:10.3389/fimmu.2018.00002)
Supplement: Supplementary file 2 [file Image_2.PDF]

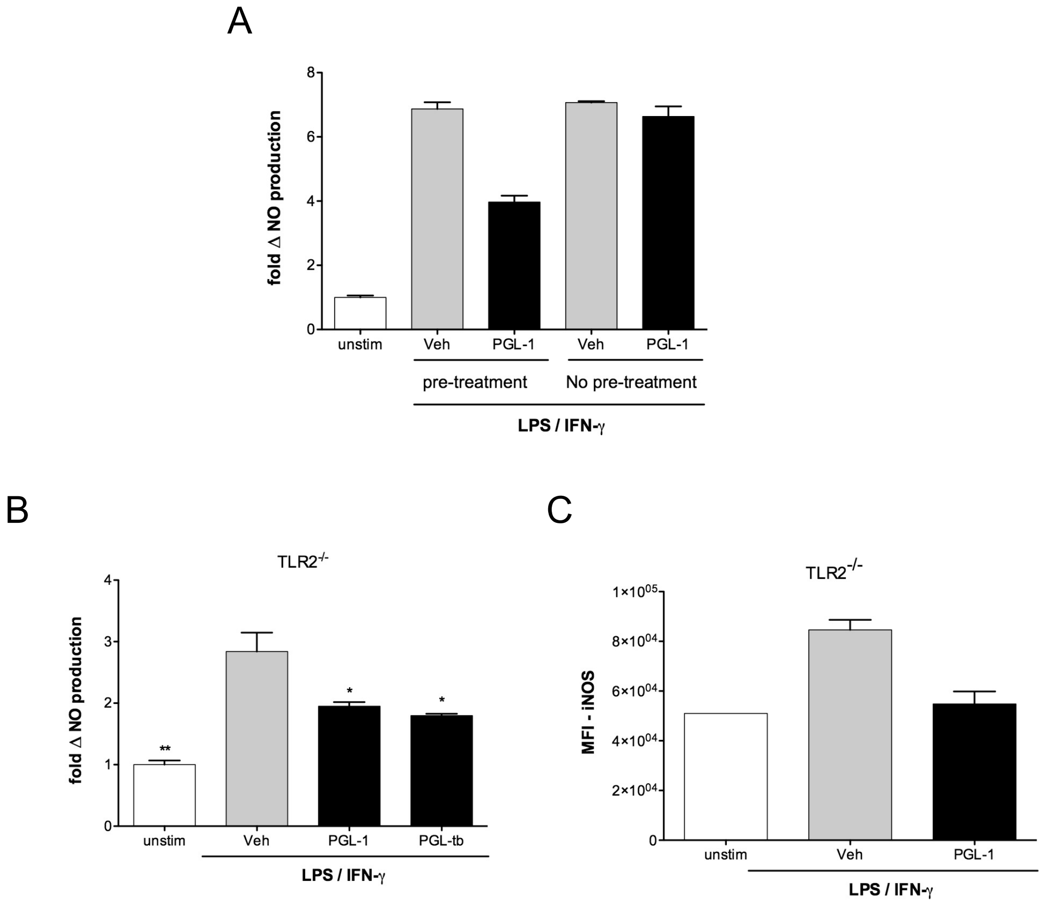

FIGURE S2. PGL-mediated inhibition of NO production by LPS/IFN-g-stimulated BMDMs requires pre-treatment and is TLR2-independent. (A) Differential production of NO by BMDMs pre-treated with increasing concentrations of PGL-1, or vehicle (Veh), for 24h prior to a 24h stimulation with 1 μg/ml LPS and 100 U/ml IFN-γ (pre-treatment), or treated with PGL-1 or vehicle at the time of stimulation with LPS/IFN-γ (no pre-treatment). Data are mean NO levels +/- SEM (n=2), expressed as fold changes relative to non-treated, non-stimulated (unstim) controls. Data are representative of two independent experiments with similar results. (B) Differential production of NO by $TLR2^{-/-}$ BMDMs pre-treated with 25 μM PGL-1, 25 μM PGL-tb, or vehicle (Veh) for 24h prior to a 24h stimulation with 1 μg/ml LPS and 100 U/ml IFN-γ. Data are mean NO levels +/- SEM (n=4), expressed as fold changes relative to non-treated, non-stimulated (unstim) controls. *P<0.05, **P<0.01, repeated measures ANOVA with Tukey post-hoc test, relative to the vehicle-treated, LPS/IFN-γ-stimulated group. (C) Differential induction of iNOS in $TLR2^{-/-}$ BMDMs exposed to 25 μM PGL-1 or vehicle (Veh) for 24h prior to a 24h stimulation with LPS/IFN-γ. Controls include non-treated, non-stimulated cells (unstim). Data are mean MFI +/- SEM (n=2). Data in (B,C) are representative of two independent experiments with similar results.
