# Supplementary material for: Mycobacterial Phenolic Glycolipids Selectively Disable TRIF-Dependent TLR4 Signaling in Macrophages
Source: Front Immunol. 2018 Jan 19;9:2. doi: 10.3389/fimmu.2018.00002 (PMC5780341; doi:10.3389/fimmu.2018.00002)
Supplement: Supplementary file 3 [file Image_3.PDF]

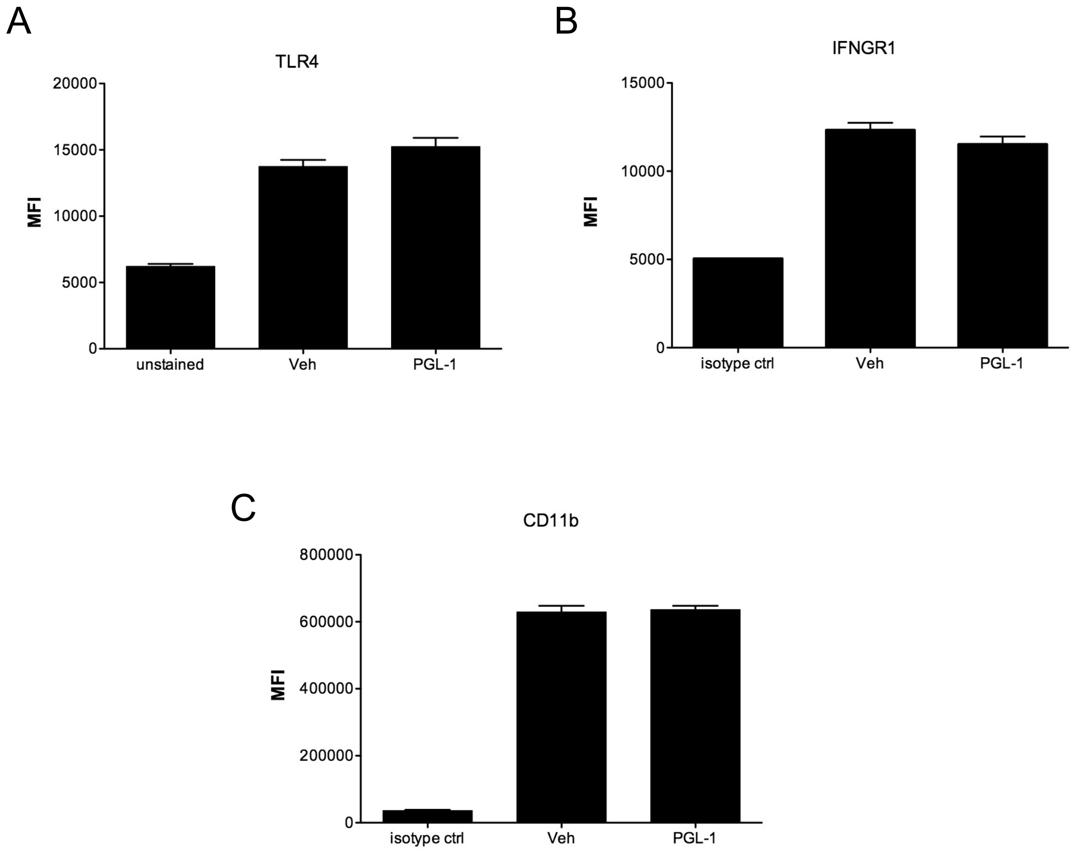

FIGURE S3. PGL-1 treatment does not affect BMDM surface expression of TLR4, IFNGR1 and CD11b. Flow cytometric analysis of the surface expression of TLR4 (A), IFNGR1 (B) and CD11b (C) by BMDM treated with 25 μM PGL-1 or vehicle (Veh) for 24h. Data are mean MFI +/- SEM of triplicates, compared to unstained or isotype controls. They are representative of two independent experiments with similar results.
