# Supplementary material for: Mycobacterial Phenolic Glycolipids Selectively Disable TRIF-Dependent TLR4 Signaling in Macrophages
Source: Front Immunol. 2018 Jan 19;9:2. doi: 10.3389/fimmu.2018.00002 (PMC5780341; doi:10.3389/fimmu.2018.00002)
Supplement: Supplementary file 4 [file Image_4.PDF]

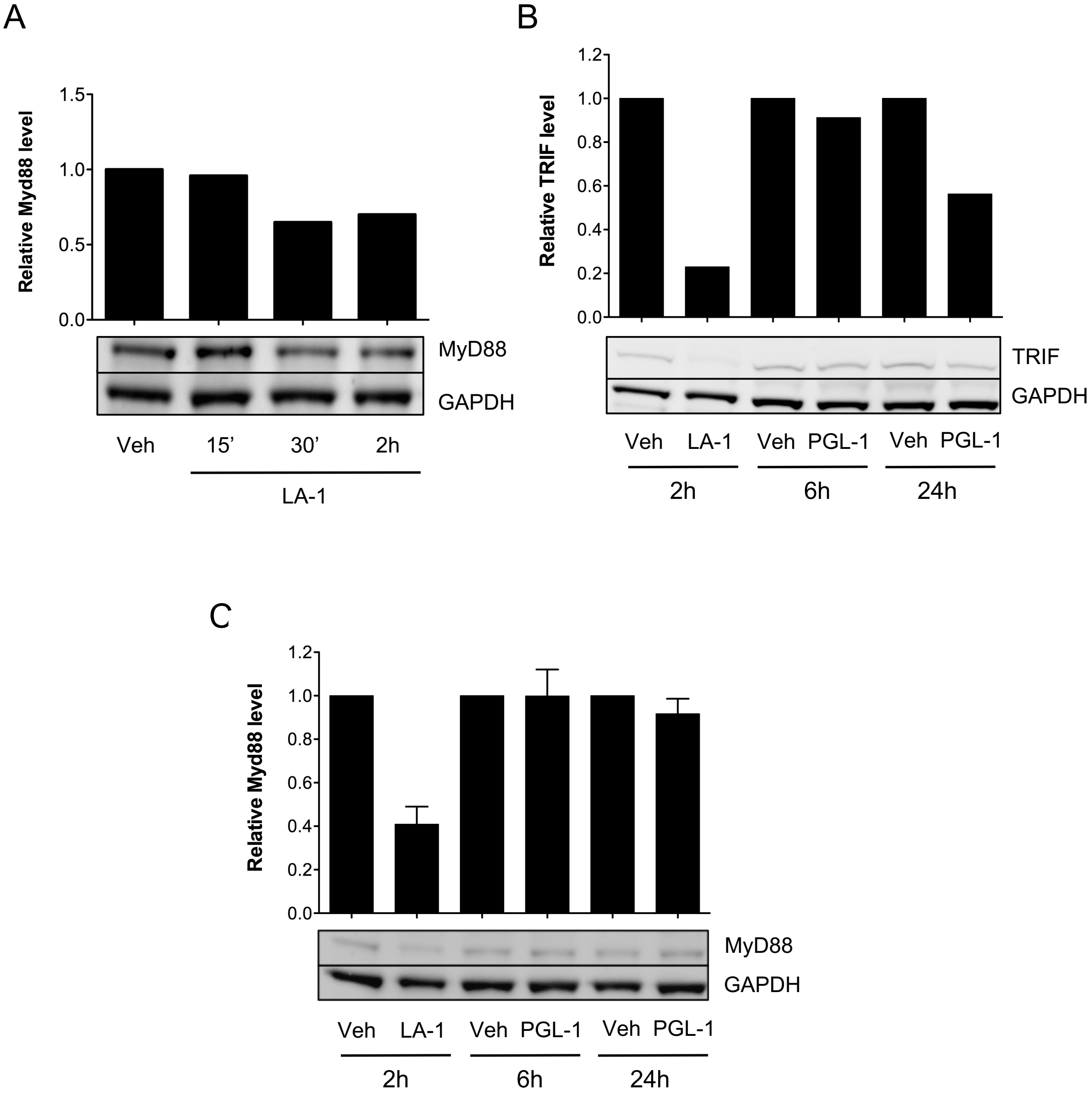

FIGURE S4. Effects of PGL-1 on MyD88 and TRIF levels in macrophages. (A) Western blot analysis of MyD88 with GAPDH as loading control, in BMDM treated with 15 µM LA-1 or DMSO vehicle (Veh) for the indicated times with relative quantification of MyD88 levels. (B) Western blot analysis of TRIF with GAPDH as loading control in THP-1 macrophages treated with 15 µM LA-1 or 25 µM PGL-1, or their respective solvents (Veh). (C) Western blot analysis of MyD88 with GAPDH as loading control, in BMDM treated with 15 µM LA-1 or 25 µM PGL-1, or their respective solvents (Veh). Mean band intensities +/- SEM from 5 independent experiments, with one representative blot picture.
