# Supplementary material for: Mycobacterial Phenolic Glycolipids Selectively Disable TRIF-Dependent TLR4 Signaling in Macrophages
Source: Front Immunol. 2018 Jan 19;9:2. doi: 10.3389/fimmu.2018.00002 (PMC5780341; doi:10.3389/fimmu.2018.00002)
Supplement: Supplementary file 5 [file Image_5.PDF]

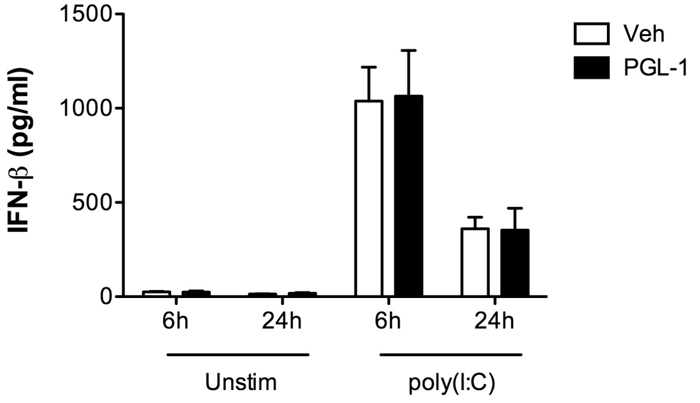

FIGURE S5. PGL-1 pre-treatment does not alter the IFN-β response of poly(I:C)-stimulated BMDMs. Production of IFN-β by BMDMs pre-treated with 25 μM PGL-1 for 24h prior to 6-24h of stimulation with 10 μg/ml poly(I:C). Data are mean cytokine concentrations from 3 biological replicates +/- SEM.
